# Vascularity of Dentine

**Published:** 1857-03

**Authors:** J. Richardson


### DENTAL REGISTER OF THE WEST.

VOL. X.—MARCH, 1857.—NO. 3.

### Original Essays and Communications.

### VASCULARITY OF DENTINE.

BY J. RICHARDSON.

Although the subject indicated at the head of this article, may seem, to those at least who entertain an *unquestioning* veneration for *authority*, to be foreclosed against further inquiry, by the somewhat emphatic announcement that "the vascularity of the teeth is now too well established to leave any room for doubt,"* yet recent ominous signs of heresy, upon this point, indicate some intolerance of a questionable dogma, and portend, if we mistake not, a somewhat different view from that proclaimed in the bull just quoted.

In protesting against this *ex parte* adjudication of the question, it is with no purpose of mere *innovation*, or idle ambition to unsettle what is esteemed by many in the profession, whose opinions are entitled to all proper deference, as a well grounded doctrine.

The inquiry into its claims upon our acceptance, will be pursued in this article, with the single purpose of arriving at as close an approximation to correct views as the nature of the evidences at our command,—in part, unavoidably, of an inferential or negative character, will admit of, asking only in their behalf a liberal and impartial estimate of their sufficiency.

* American edition of Fox, by C. A. Harris, p. 63.

To those who have taken the pains to make the subject under consideration one of thoughtful study, it may seem somewhat unaccountable that concurrently with the progressive step taken from the *vital theory* of decay, of the older writers, to that of the *chemical*, as received with great unanimity at the present day, there should not have been a corresponding change, or *advance* of opinion, in regard to those assumed properties of dentine in which the former had its origin, and out of which it naturally and unavoidably grew. The belief that *caries* was the result of certain vital changes effected in the constitution of the teeth, through some modification or other of inflammation, and that these changes were effected primarily within their texture through the agency of intrinsic causes, had at least a reasonable show of consistency ; for it flowed naturally from a belief of its vascularity. Advocating the doctrine of a circulation in teeth, analogous with that of bone, they held, with much propriety, that inasmuch as they were identical in respect to their general vital conditions and properties, that whatever agencies were competent to effect a disorganizing process in one, were equally capable of producing it in the other. The conclusion had all the force and plausibility of a logical deduction. Hence Mr. Thomas Bell, and others, advocating the vital theory of decay, came to regard *inflammation* as the proximate cause of dental caries, because the same condition, in some form or other, was observed to prevail in a similar affection of bones generally.

Now we think it may be justly asserted, that the position of these writers was perfectly defensible on the assumed grounds of the vascularity of dentine, and that a continued adhesion to this latter view, will drive us to the extremity, either of controverting the well settled principle in pathology that *all vascularized tissues* are capable, under some circumstances, of suffering degeneration and death from the primary operation of *intrinsic* causes, or by a retrograde movement of falling back upon exploded theories, and admitting that *some-*

*times* at least, caries may result from inflammation acting primarily upon the dentine. But we are saved from this humiliating apostacy from the modern faith, by the assurance from Prof. Harris, and which commends itself fully to our acceptance, that "a thorough investigation of this subject ought to convince any one, that caries always commences externally." But to have rendered the opinion more complete, he might have added, as we volunteer to do by way of supplement, that a like thorough investigation of the subject ought to convince any one that the adoption of this opinion and the advocacy of the vascularity of the teeth involves, what we think may be made to appear, a very transparent antagonism. Their respective signification may be stated under two propositions, thus :

1st. According to the author's own showing, *inflammation*, as a primary affection of dentine, resulting in its death and decomposition, cannot, and never does *precede* the action of *external chemical agents* in the production of caries. In summing up the evidences against the "vital" theory, he asks, "If inflammation of their dentinal structure, then, is *not* the cause of the affection, how is the disease produced?" Following up the subject, he continues, "But it may be asked if caries be produced by the action of external corrosive agents, how is it that the disease sometimes commences within the dentinal structure of a tooth, and makes considerable progress there before any indications of its existence are observed externally ? We answer that it *never does commence there.*"

This opinion, defended by evidences that lead fairly to a conviction of its correctness, if it has any special significance, whatever, means simply this, that inasmuch as the conditions of disease never prevail in the manner stated by Bell and others, that therefore the dentinal structure, by virtue of its mode and measure of endowment, is *incapable* of such alterations or modification of its normal condition.

Upon an equally logical exposition of the doctrine of its

*vascularity*, the antagonism referred to will appear in the statement of proposition

2nd. It may, upon adequate authority, be stated as a well settled principle, that although the *absence of vessels* in any part does not furnish unquestionable proof of its *inaptitude* for inflammation (though the weight of authority inclines to an affirmative opinion), yet the converse is held to be true,—that their *presence* implies, always, a condition favorable to its development. Measured by this standard, therefore, dentine, on the presumption of its vascularity, becomes obnoxious to the operation of the same laws that determine the issue of disease in other organs analogously constituted, modified in some degree, doubtless, by peculiarities of structure, but certainly not to the extent of complete immunity therefrom. This view, therefore, authorizes and enforces the belief that the dentine of teeth being vascular, may suffer degeneration and death from the operation of internal as well as external causes, in advance of chemical agents acting from without.

It need hardly be insisted, we think, that this presentation of the subject places these two propositions in such an attitude of irreconcilable hostility to each other, that it would seem irrational and unintelligible for the same mind to accept one without discarding the other. Our couclusion therefore is, that if the *chemical theory* furnishes the only acceptable expose of the causes and nature of decay compatible with common observation and a fair interpretation of its phenomena, it is palpably illogical and unwarrantable to claim for dentine, in regard to its vascularity, what necessarily falsifies the theory and discredits every well-attested fact upon which it is predicated. The true import of the received opinion, concerning the causes of decay, is plainly this, that the dentine of teeth being *non-vascular*, is incapable of suffering primary lesions from the operation of *intrinsic* causes, and that this *incapacity*, is the only rational and consistent basis on which it is possible, agreeably to ascertained laws, to construct a theory referring the origin of caries to the *exclusive* action of external chemical agents.

We pass to the consideration of other evidences, based upon the results of microscopic examinations of the anatomical structure of dentine. And here it will appear evident that we have been receiving largely upon trust, when it is stated that the dentine of human teeth at least, has never been successfully *demonstrated* to be vascular, as an ordinary or common condition of its structure, notwithstanding it has been made the subject of repeated, careful and critical research, under the management of unquestionable skill and experience.

What then are the main structural characteristics of dentine? It may be stated briefly that, according to most microscopic anatomists, it consists in part of minute *tubes*, or *hollow fibres*, provided with special parietes, and containing, according to Müller and Retzius, "granular masses of inorganic matter." Mr. Alexander Nasmyth, however, whom Prof. Harris pronounces "equally distinguished as an odontologist," has demonstrated by a series of "beautiful and highly interesting experiments," that the *hollow tubes* of Owen, Tomes, Koelliker, and others, are not such, but "*solid fibres*, composed of a series of little masses, succeeding each other in a linear direction, like so many beads collected on a string." Besides these tubes or fibres, dentine is composed of a granular or interfibrous substance, whose positive conditions are not very well understood, or at least furnish subject-matter for some discrepancy of opinion. "Each of these granules," says Prof. Harris, "if indeed the interfibrous tissue is made up of them, is enclosed in a delicate investment of animal matter, upon which exceedingly minute vessels are, no doubt, distributed." This conclusion of the author comes in so questionable a shape as a naked and unsupported assumption, that we will be held excusable for receiving it at a discount, until demonstrations, at least, shall surround what must at present be esteemed an unsupported conjecture, with some higher degree of probability.

There are other structural features of dentine not especially concerned in the treatment of this subject.

The dental tubuli, viewed generally as the natural channels for blood-vessels, considered with reference to their *calibre*, furnish a presumption amounting almost to positive proof, of their inaptitude for the purpose usually assigned them. The most reliable and expert microscopists have demonstrated their largest diameters to be only about 1-10,000th of an inch, decreasing in size as they pass out from the pulp-cavity, until their peripheral diameters become too small for computation. To claim that these tubes or canals are traversed by vessels charged with blood, containing either *red* or *colorless corpuscles*, is equivalent to assuming a physical impossibility; for, having an average diameter of about 1-3200th of an inch, they represent a capacity more than three times greater in respect to diameter, than the tubuli. This estimate excludes the parietes of the vessels, increasing still more the disproportion.

But it is claimed that these corpuscles of the blood may be excluded, and that vessels are existant conveying only the "liquor sanguinis," or thinner portions of the blood. After detailing some anomolous specimens of vascular dentine, Prof. Harris remarks, "Now, if vessels have been detected in dentine in so many instances, it seems more than probable that they are always present, though too small and attenuated to convey anything but the thinnest and most serous part of the blood."* Dr. Carpenter, whose estimate of any physiological question is entitled to great weight, thus disposes of this view—"The opinion was long entertained that there are vessels adapted to supply the white or colorless tissues, carrying from the arteries the 'liquor sanguinis,' and leaving the corpuscles behind through inability to receive them. But such a supposition is altogether groundless."† The opinion referred to by the author sprang from the generally adopted no-

* Principles and Practice of Dental Surgery, p. 51.

† Principles of Human Physiology, p. 299.

tion, at an earlier period, that the nutrition of any organ could only be maintained by blood-vessels being intimately distributed to all its parts,—an idea he pronounces " entirely gratuitous." But again, the supposition that vessels are present conveying only the " thinnest and most serous parts of the blood," is rendered improbable for the following reason: Before their dimensions can be accommodated by the dental tubuli, their diameters must be reduced to fully *one-third* the average diameter of a common capillary, which, according to Müller and Weber, measure but 1-3100th of an inch, three times larger than the tubes into which it is proposed to place them,—provided these tubes are not, according to Mr. Nasmyth, "*solid fibres.*"

We wish it remembered in this connexion, that in our estimate of these relative measurements, so far as it concerns the tubuli, reference has been had *only* to their *largest diameters.* If at this point there is so great a want of correspondence between the diameter of the latter and the blood corpuscles and capillaries, upon what grounds, we ask, can we assume the existence of vessels at remoter distances from the pulp-cavity, where, in the language of Dr. Carpenter, they become " immeasurably fine." The assumption implies, in plain English, an absurdity too palpable for serious entertainment.

Dr. Horner, in his treatise on Anatomy and Histology, in an article on the evolution of the teeth, quoting from the observations of Hunter and others, furnishes the following paragraph: " The adhesion of the pulp to the new formed bone, is such as to require some slight force to separate them ; but this may be done without rupturing materially either the one or the other ; *their surfaces which were in contact are smooth,* neither is there any evidence of a vascular communication between them."

If the fact conveyed in this extract, italicised by us, is one upon which we can rely as authentic, it confirms the observation of the experimenter, and excludes the idea of a vascular connection between the pulp and tubuli ; for otherwise the denuding of the bone would be followed by some exudation from the open mouths of the torn vessels, besides presenting some appearances of lacerated membranes ; for if vessels pass from the pulp-cavity, their parietes, according to all analogy, must be derived from the lining membrane of the pulp, the violent disruption of which would scarcely leave the surface in the condition mentioned.

Although Mr. Tomes relates some instances of vascular dentine, he recognizes them as exceptional and not as constant conditions of its structure. "We have, in many instances, canals for vessels traversing it, just as we have Haversian canals perforating bone. In the teeth of man these vascular canals are never numerous, and occur only in a few specimens. I have seen eight or ten sections only in which the dentine is vascular." The canals here spoken of are distinct from the tubuli, for he says, "In each instance many of the neighboring dentinal tubes have terminated by open mouths on the surface of the canal, and in one case the canal was *clothed on one side with a thin layer of cementum.*"*

The observation with which this paragraph closes, contains a suggestion of much importance in this discussion. To what extent this abnormal and erratic development of cementum, (which is generally *vascular*), may become incorporated with the proper texture of dentine, and to that extent determine its vascularity in particular instances (as in those related by Tomes and Harris), cannot be positively determined, but there are probabilities connected with it that make the inquiry one that may be profitably pursued. Anomalous specimens, in which vessels have been distinctly *demonstrated* in dentine, constitute the "head and front" of the evidence upon which the *facile* conclusion is sought to be engrafted, that they are but typical of its common condition: these aberrations explained to the exclusion of the hypothesis attached, and the whole defense of the doctrine is reduced to a "forlorn hope."

Mr. Tomes observes, "The cement and dentine possess

* Dental Physiology and Surgery, p. 49.

so many properties in common, and are often so like each other, that in some specimens *it is difficult to determine to which of the two tissues the various parts belong.* Thus, in the cementum, we find tubuli terminating in open mouths upon a vascular surface, such as the surface of the root of a tooth, while in the dentine we observe the presence of cells with radiating tubes; indeed we see the dentinal tubuli taking the form of cells with tubular branchings. In the tooth of the kangaroo, the two tissues are fairly mixed." "Some few specimens taken from the fangs of human teeth, exhibit the two tissues fairly intermingled."*

Observe in this connection that all those cases of vascular dentine mentioned by Mr. Tomes, were confined to the *roots* of teeth. "In those instances of vascular canals traversing human dentine, which have come under my observation, their direction has been from the pulp cavity towards the surface of the roots of the teeth."†

It is a reasonable conjecture, therefore, that with the intermingling of these tissues, the dentine to some extent becomes impressed with the same properties common to cementum, vascular as well as structural, and to this mode of abnormal development we must for the present refer those anomolous specimens that have, with some ostentation, been paraded as furnishing proof of a uniform vascular circulation in teeth. As the condition alluded to is but *accidental,* we may regard these instances of vascular dentine as *departures* from, rather than as types of, its common organization.

We do not think it necessary to multiply authorities upon this subject. A single extract from Dr. Carpenter's recent work, entitled "The Microscope and its Revelations," will sufficiently indicate this distinguished physiologist's views. Alluding to the diameter of the dental tubuli, he says, "It is impossible that even the largest of them can receive blood, as their diameter is far less than that of the blood-discs; but

* Dental Physiology and Surgery, p. 63.

† Id., p. 50.

it is probable that, like the canaliculi of bone, they may absorb nutrient matter from the vascular surface upon which their inner extremities open."

The vascularity of dentine has been inferred from the occasional appearance of reddened discoloration of its substance, noticed usually in connexion with some diseased condition of the teeth. The same also has been observed as a *post mortem* phenomenon, in the teeth of those who have died from suffocation. In jaundice, too, the teeth sometimes acquire the peculiar hue characteristic of that disease.

The explanation of these appearances is now very generally, as well as properly referred, *not to a circulation of red corpuscles*, but to some original affection of the blood itself, within the vessels of the pulp, eventuating in the disorganization of these corpuscles, the coloring matter of which, in a state of solution, is carried into the tubuli, infusing itself through the entire substance of the dentine. It is impossible to account for the appearances spoken of, on the supposition of the circulation of red corpuscles in their normal or unruptured state; for, keeping in view the diameter of the tubuli, they could only enter, if at all, distorted and in "single file," in which condition they do not impart any coloring, this being effected *only* by their *aggregation.* Hence it is that the capillaries, in their ordinary condition, when exhibited singly, are colorless, admitting as they do but a single row of corpuscles. It is true that they may be reddened by *injection*, for their parietes are dilatable and admit of unnatural *accumulations*, but in dentine, the tubuli, surrounded by casements of unyielding bone, utterly excludes any such modification of their diameters. This view, therefore, renders the explanation we have given the only admissible one.

It may be asked, if dentine is not supplied with blood-vessels conveying nutritive matter to it, how is its *vitality maintained?* We are aware that it is not an uncommon opinion that a part, to be maintained in its nutrition, must have the elements of the blood carried by vessels distributed intimately through every portion of it,—an opinion presupposing that the *vessels* themselves are *actively* concerned in the process of its growth and renewal. We need hardly say that this idea is erroneous. Nutrition, everywhere, is an *extra-vascular act*, and it is immaterial whether the tissue to be nourished receives nutrient matter *directly* from the blood-vessels, or is conveyed to distant parts by the process of *imbibition*; the same *completeness* of the act being accomplished in either case as the tissue, from its high or low grade of organization, may demand an active circulation, or otherwise. Instances of non-vascular tissues are not wanting, nourished by the process of imbibition. Much of the substance of common bone is vitalized in this way. Those portions of it denominated the "Haversian ossicles," are not supplied with vessels, but absorb nutriment from the Haversian canals, through different systems of tubes opening upon these vascular channels, and forming intimate communications with each other and with the lacunæ. We may regard these portions of bone as occupying, in respect to their nutrition, the same relation to the canals of Haver that dentine does to the pulp-cavity.

We have also familiar illustrations of this method of nutrition in the epidermis; the lachrymal bones of the orbit, the cornea, crystalline lens, and vitreous humor of the eye, the turbinated bones of the nose, the articular cartilages, etc., etc.

Now, in the light of such evidences as we have but imperfectly presented in the discussion of this subject, omitting many additional considerations that might have been offered, we believe, without *conscious* arrogance, that the question of the vascularity of the teeth is *not* "too well established to admit of a *doubt*."

In conclusion, we may be allowed to suggest, that in respect to these organs, there is nothing either in their uses or the circumstances of their being, that demands a more active or complicated organism than that generally imputed to *non-*

*vascular* tissues. On the contrary, any other condition would be incompatible with their purposes and well-being, by continually subjecting them to casualities that would not only endanger their security and defeat their usefulness, but render them, under the operation of common causes, both internal and extrinsic, afflictive beyond endurance.

